# What the eyes, confidence, and partner’s identity can tell about change of mind

**DOI:** 10.1093/nc/niae018

**Published:** 2024-05-07

**Authors:** Rémi Sanchez, Anne-Catherine Tomei, Pascal Mamassian, Manuel Vidal, Andrea Desantis

**Affiliations:** Département Traitement de l’Information et Systèmes, ONERA, Salon-de-Provence F-13661, France; Institut de Neurosciences de la Timone (UMR 7289), CNRS and Aix-Marseille Université, Marseille F-13005, France; Département Traitement de l’Information et Systèmes, ONERA, Salon-de-Provence F-13661, France; Institut de Neurosciences de la Timone (UMR 7289), CNRS and Aix-Marseille Université, Marseille F-13005, France; Laboratoire des systèmes perceptifs, Département d’études cognitives, École normale supérieure, PSL University, CNRS, Paris F-75005, France; Institut de Neurosciences de la Timone (UMR 7289), CNRS and Aix-Marseille Université, Marseille F-13005, France; Département Traitement de l’Information et Systèmes, ONERA, Salon-de-Provence F-13661, France; Institut de Neurosciences de la Timone (UMR 7289), CNRS and Aix-Marseille Université, Marseille F-13005, France; Integrative Neuroscience and Cognition Center (UMR 8002), CNRS and Université Paris Cité, Paris F-75006, France

**Keywords:** perceptual confidence, pupillary response, metacognition, human–partner interaction, change of mind

## Abstract

**Perceptual confidence reflects the ability to evaluate the evidence that supports perceptual decisions. It is thought to play a critical role in guiding decision-making. However, only a few empirical studies have actually investigated the function of perceptual confidence. To address this issue, we designed a perceptual task in which participants provided a confidence judgment on the accuracy of their perceptual decision. Then, they viewed the response of a machine or human partner, and they were instructed to decide whether to keep or change their initial response. We observed that confidence predicted participants’ changes of mind more than task difficulty and perceptual accuracy. Additionally, interacting with a machine, compared to a human, decreased confidence and increased participants tendency to change their initial decision, suggesting that both confidence and changes of mind are influenced by contextual factors, such as the identity of a partner. Finally, variations in confidence judgments but not change of mind were correlated with pre-response pupil dynamics, indicating that arousal changes are linked to confidence computations. This study contributes to our understanding of the factors influencing confidence and changes of mind and also evaluates the possibility of using pupil dynamics as a proxy of confidence**.

## Introduction

Every decision we make is accompanied by a sense of confidence. For instance, when biking at night we might feel less confident about the speed and behavior of incoming traffic, and this leads us to reduce our speed. Confidence is regarded as the ability to evaluate the quality of our mental representations and decisions (Fleming and Daw 2017). Recent theoretical accounts have suggested that confidence is critical for learning (Meyniel et al. 2015, Guggenmos et al. 2016) as well as to enable adaptive behavior under uncertainty (Soltani and Izquierdo 2019). For instance, it is regarded as an internal feedback signal guiding learning strategies (Bjork et al. 2013) and memory “offloading” activities, such as writing down the items to buy in a shopping list (Risko and Gilbert 2016). Moreover, it is also thought to be critical for human–machine interactions: poor confidence in the decisions of the machine can lead to a lack of cooperation and coordination between the human and the machine, which would hinder the effectiveness of the interaction (Zhang et al. 2020, Dafoe et al. 2021, Steyvers and Kumar 2023).

However, only a few empirical studies have actually investigated the role of perceptual confidence in behavior and decision-making. The difficulty in linking perceptual confidence to behavior relies on the fact that decision accuracy and confidence are strongly correlated: the higher the quality of sensory evidence, the higher the accuracy and also the confidence in one’s perceptual decisions. Hence, it remains unclear whether it is confidence or the quality of sensory evidence that influences upcoming behavior and individuals’ decision strategies (Desender et al. 2018). Recent research started to tackle this issue and highlighted the contribution of perceptual confidence in information-seeking behavior (Desender et al. 2018, Schulz et al. 2023) and in change of mind (Fleming et al. 2018, Rollwage et al. 2020, Pescetelli et al. 2021). Other research also showed that confidence is particularly beneficial during collective decision-making (Bang et al. 2014). For instance, perceptual confidence is communicated verbally by participants in order to interact optimally (Bahrami et al. 2010).

The present study aimed at investigating whether perceptual confidence, rather than first-order representations of sensory evidence (i.e. measured by the accuracy of perceptual decisions), regulates the interaction with a partner. To address this question, participants completed a perceptual discrimination task with a partner. They were presented with two random dot kinematograms (RDK), one to the left and the other to the right of a central fixation point. Participants reported which RDK had a coherent motion direction closer to the vertical axis, and indicated their confidence in their perceptual decisions. Subsequently, participants viewed the perceptual decision of a (fictitious) partner. Notably, in separate blocks, they were presented with the responses of either another human participant or a machine. They were told that the human/machine partner completed the same task and that their responses were replayed. However, in reality, both partners were fictitious and were programmed by the experimenter to achieve the same level of performance. Regarding to the machine partner, participants were told that it was a machine learning algorithm and that just like human participants it was not infallible. After viewing the partner’s response, participants could keep or change their initial perceptual decision. They were explicitly instructed to use the response of their partner if they thought this could improve their performance.

We hypothesized that if perceptual confidence, rather than first-order representations of sensory evidence (i.e. measured by the accuracy of perceptual decisions), guides the interaction with a partner, then multilevel regression analyses should reveal that participants’ decisions to change their initial response will be more strongly predicted by their subjective confidence rather than by their accuracy in perceptual decisions.

To bolster these conclusions, we manipulated contextual factors with the aim of inducing fluctuations in confidence without impacting perceptual performance (i.e. accuracy). As mentioned earlier, we manipulated the partner’s identity with the expectation that this manipulation might bias confidence judgments while leaving accuracy unaffected, and also to assess whether the partner’s identity influences participants’ interaction strategies. In fact, recent studies have demonstrated that machines can evoke either overconfidence (Booth et al. 2017, Booth 2020) or mistrust (Nicodeme 2020, Seth and Kishore 2020, Lee and Rich 2021) depending on the context. In addition, we manipulated the variability of the coherent dot motion, since previous research showed that stimulus variability can distinctly impact confidence and accuracy. For instance, high variability leads to a decrease in confidence while low variability increases confidence (Desender et al. 2018). In our task, dots could move coherently in a given direction with either high or low variance. During a preliminary calibration phase, we ensured that perceptual performance remained constant between high- and low-variance conditions. Based on these manipulations, we hypothesized that confidence, but not accuracy, would be influenced by stimulus variability and partner’s identity. In addition, this change in confidence would be accompanied by participants’ tendency to change their initial decisions.

Finally, we also investigated the relationship between pupil dilation and confidence. There is growing interest in the field of neuroergonomics to monitor in real-time different cognitive states (e.g. attention, vigilance, etc.) of operators while they interact with technology (Dehais et al. 2017, 2020, Gramann et al. 2017). This monitoring is especially valuable when explicit responses from operators during the task are not available (e.g. when operators are required to supervise the activity of an automated system). Metacognition and confidence are recognized as crucial cognitive processes to monitor, since they play a critical role in optimal human–machine interactions (Lee and Moray 1992, Parasuraman et al. 1993). Past research established a link between pupil size and decision uncertainty (Lempert et al. 2015, Urai et al. 2017, Balsdon et al. 2020). Consequently, pupil dilation holds the potential to offer valuable insights into individuals’ confidence without relying on overt behavior. In line with these notions, this study aimed to provide evidence that pupil dilation can serve as a predictive marker for individuals’ confidence judgments.

Unlike prior studies that explored post-decisional pupil changes linked to decision uncertainty, we specifically investigated pre-response pupil fluctuations that might predict confidence judgments. The ability to predict decision uncertainty from pupil dilation during when sensory information is processed is important, since it allows monitoring individuals’ confidence and uncertainty continuously in the absence of overt behavior. Furthermore, the link between confidence and pupil dilation can provide insight into the relationship between confidence and physiological arousal. Recent studies have established a connection between arousal and uncertainty (O’Connell and Kelly 2021). In the current study, to highlight changes in pupil-linked arousal, we specifically focused on pupil dilation velocity. This choice is informed by recent studies indicating that the temporal derivative of pupil size more closely reflects the dynamics of arousal fluctuation (Reimer et al. 2014, 2016, Okun et al. 2019, Crombie et al. 2021).

The analysis of pupil dilation also prompted an investigation into the potential link between confidence and eye blinks. The motivation for analyzing eye blinks was 2-fold: firstly, eye blinks may impact pupil dilation (Lee et al. 2021), and thus, we aimed to remove this potential confound. Secondly, blinks are thought to play a role in regulating attention and arousal. Individuals tend to blink more frequently when engaged in tasks that require sustained attention, and eye blinks have been used as an index of an individual’s level of arousal, alertness, or even fatigue (Oken et al. 2006, Maffei and Angrilli 2018, Gavas et al. 2020).

In summary, we observed that participants’ confidence more reliably predicted their decisions to change or keep their initial perceptual response than did accuracy and task difficulty. Additionally, when participants believed they were interacting with a machine rather than a human, their confidence decreased and their tendency to change their initial perceptual response increased. Statistical analyses suggested that the partner’s identity impacted confidence and change of mind independently. Furthermore, pupil changes prior to the response correlated with observers’ confidence, suggesting that pupil variation could be used as an online proxy for confidence in the absence of an explicit response. Finally, eye blinks are also correlated with confidence judgments. This study contributes to our understanding of the role of confidence and contextual factors (i.e. partner’s identity) on change of mind and also evaluates the possibility of using pupil dynamics as a proxy of confidence.

## Materials and methods

### Participants

Based on similar experiments investigating confidence (cf. de Gardelle et al. 2015; Desender et al. 2019), the sample size was set to 14 adult participants that fully completed the experiment. In total, 21 adults were recruited on a voluntary basis and received a financial compensation of 10€ per hour. Seven were not included in the sample size, six participants did not complete the task due to technical problems with the eyelink or difficulties with maintaining fixation during stimulus presentation, and one participant judged s/he was very confident in all her/his responses. The remaining 14 adults (9 female, range of 19–36 years of age) completed the experiment and were analyzed. All participants had normal or corrected to normal vision, and were naïve to the hypothesis under investigation. This study was conducted in accordance with the requirements of the Declaration of Helsinki and approved by the Ethics Committee of the Université Paris Descartes. The experiment lasted approximately 2 h and 30 min in total, including instructions, breaks, training, and eye-tracker calibration.

### Apparatus

Stimuli were presented on an LCD monitor (Samsung 2232RZ, 47 cm wide) with a refresh rate of 100 Hz and a resolution of 1680 × 1050 pixels. Stimulus presentation and data collection were performed using MATLAB with the Psychophysics Toolbox (Brainard 1997) and the Eyelink Toolbox (Cornelissen et al. 2002). Viewing was binocular and movements of the right eye were monitored with an EyeLink 1000 Plus (SR Research, Mississauga, ON, Canada) at a sampling rate of 1000 Hz. Head movements were restrained with a chinrest located 70 cm from the screen.

### Stimuli

Stimuli were two RDKs presented simultaneously to the left and right of a central fixation point (a white empty circle of 0.3° visual angle diameter) at an eccentricity of 4° from fixation. RDKs consisted of 70 white dots displayed within a circular aperture of 3° visual angle diameter. Each dot had a diameter of 5 pixels (0.114° visual angle). Dots of each RDK moved coherently (5°/s speed) upward with a specific tilt angle from the vertical axis that was determined for each participant in a preliminary experiment (see below). The dot lifetime was set to 300 ms after which it was erased and then displayed in the symmetric location relative to the center of the circular aperture.

### Procedure

#### Preliminary experiment

**Figure 1.**
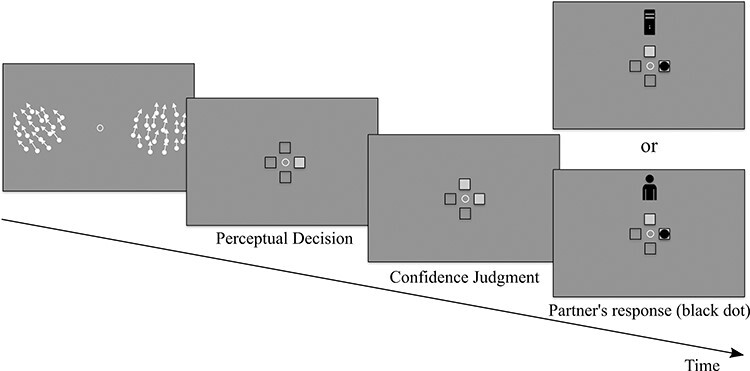
Schematic illustration of a trial. The two RDKs appeared 500 ms after a display screen that contained only a central fixation point. The dot motion was presented for 1200 ms. Thereafter, the RDKs disappeared and four squares were presented indicating the participants to report which RDK contained the dot motion that was closer to the vertical axis by pressing the left or right arrow. Participants could then report their confidence before viewing the response given by their partner. After the presentation of the partner’s response, they could choose whether to keep or change their initial perceptual judgments. There was no confidence associated with the partner’s response when it was displayed. Participants reported their second response using the same key mapping as for their first response

Before starting the main experiment, we assessed participants’ tilt discrimination thresholds with a preliminary experiment. In this experiment, participants were presented with two RDKs, and they were required to report in which one of the two (i.e. the left or the right one) dots moved coherently closer to the vertical axis. No confidence judgment was required, and the participants were not presented with the responses of the partner during this phase. Specifically, each trial started with the presentation of a fixation point. Two RDKs were displayed on either side of fixation, 500 ms after fixation onset, and for a duration of 1200 ms. The dots on the left RDK moved upward with a slight tilt toward the left, while the dots on the right RDK moved upward with a slight tilt toward the right. While keeping their gaze on the fixation, participants had to report in which RDK (left or right) the global motion moved closer to the vertical axis, by pressing the left or right arrow key of a keyboard with their right hand. Participants were told that accuracy was more important than rapidity when reporting their perceptual decisions. If fixation was broken or a blink occurred while the stimulus was displayed, an error message appeared on the screen and the trial was interrupted. Participants responded only after the RDKs disappeared and four unfilled black squares (with a side length of 0.3° visual angle) appeared around fixation (eccentricity of 1.5° visual angle) in a cross-like shape (i.e. one square above, one below, one to the left, and one to the right of fixation, see Fig. 1). Participants’ responses were shown with the corresponding square filled with a light gray color.

The calibration to the participants’ discrimination threshold consisted in controlling the tilt angle of the dot motion direction. In each trial, we varied the tilt angle of one RDK while keeping the tilt of the other RDK at 40° (i.e. the reference). The change of tilt of each RDK was controlled by two interleaved staircases one starting with a difference of 15° and the other with a difference of 30° from the reference value (i.e. 40°). Following a correct response, the tilt difference between the left and right RDK decreased in the subsequent trial, making the discrimination task slightly more challenging. Conversely, an incorrect response led to an increased tilt difference, making the task slightly easier in the next trial. The size of this increment/decrement was controlled by an accelerated stochastic approximation algorithm (Kesten 1958) set to converge at the tilt difference that supported 75% accuracy. This method involves adapting the increment/decrement size on a trial-by-trial basis, rather than using fixed step values. It is typically employed to rapidly determine individuals’ thresholds. With this approach, the stimulus intensity in each trial is determined based on several factors, including the participant’s accuracy, the number of trials already completed, and the desired threshold. Each staircase stopped once the convergence level was reached. The convergence values reached by the two staircases controlling the left (and right) RDK were averaged and used as the participant’s tilt discrimination threshold for the left (and right) RDK. Importantly, in the preliminary experiment, we also manipulated the variability (standard deviation of the Gaussian distribution) of the dot motion directions. Dots could move coherently in the specified direction with a small (i.e. 3°) or a large variability (i.e. 15°). Separate tilt discrimination thresholds were obtained for these two variability levels. In sum, the preliminary experiment allowed estimating, for each participant, four tilt discrimination thresholds: 2 sides (left and right) × 2 variabilities (high and low).

#### Main experiment

Participants completed the same tilt discrimination task described above. The tilt difference between left and right RDKs could take one of three possible values: 70%, 100%, or 140% of the tilt discrimination threshold determined in the preliminary experiment. Hence, participants completed the task under three difficulty levels: hard (70% of threshold), intermediate (100% of threshold), and easy (140% of threshold). Finally, as for the preliminary experiment, we manipulated the variability of coherent motion, i.e. dots could move coherently with a variability of 3° or 15°. We used different variability values and difficulty levels to prompt individuals to vary their confidence judgments.

After reporting the RDK with the dot motion direction closer to the vertical axis, participants indicated their confidence on a 2-point scale, i.e. whether they were confident or not about their response, by pressing the up or down arrows of the keyboard, for “I am sure, it was correct” and “I am not sure,” respectively. Similarly, to the perceptual decisions, participants’ confidence judgment was shown by filling the corresponding upper or lower square (see Fig. 1). Confidence judgments were followed by the presentation of the perceptual response of a machine or human partner. To remind the partner who they were interacting with, a black icon of a computer or human was displayed on top of the fixation. The partner’s judgment was presented with a black circle (0.3° visual angle diameter) appearing within the square corresponding to the perceptual judgment of the partner (left/right). Note that the partner did not provide any confidence report. After visualizing the partner’s judgments, participants decided whether to keep or change their initial perceptual response. They were instructed to use the response of their partner if they thought this could improve their discrimination accuracy. They were told that both the human and machine partner viewed the same stimuli and their answers were stored and replayed. They were also told that the machine partner was a machine learning algorithm and just like a human participant it was not infallible. In reality, partners’ responses had been programmed by the experimenter and both partners were set to achieve the same performance accuracy. In particular, they were programmed to have 80%, 90%, and 100% accuracy for the hard, intermediate, and easy difficulty level, respectively. Hence, partners’ accuracy was programmed to be higher than participants’ theoretical accuracy (75% threshold in the preliminary experiment). Human and machine partner trials were divided into four blocks and were presented alternately with the order of presentation counterbalanced across participants. Each block consisted of 144 trials, with Variability and Difficulty trials being randomized and equiprobable within each block. In total, participants completed 576 trials (48 trials × 3 difficulties × 2 variabilities × 2 partners).

Participants were instructed to maintain fixation and to not blink both during RDKs presentation and before their perceptual response. Fixation was considered broken when the gaze moved more than 1.25° away from the fixation center. If a fixation break or a blink occurred, the trial was interrupted, an error message was displayed (“please fixate”), and the same trial repeated at the end of the block.

### Behavioral analyses

Logistic mixed effect models were employed to fit accuracy, confidence, and switch responses. Model fitting was performed using the “lme4” package (Bates et al. 2015) and the lmerTest package (Kuznetsova et al. 2017) in R (Team, R. C. 2018). Factors with two levels were coded using sum contrasts (Schad et al. 2020). The models were adjusted to accommodate convergence and singularity issues. Specifically, the parsimony principle guided the selection of random effects (Bates et al. 2018). Indeed, for some analyses, the inclusion of the maximal random structure led to convergence failures. In these cases, we performed a Principal Component Analysis (PCA) to isolate the random effects that contributed the least to model fitting and we removed them from the final model. The significance of the fixed effects was determined using type-II Wald tests with the “car” package in R (Fox and Weisberg 2011). Post-hoc comparisons were performed with the package “emmeans” (Searle 1980). When required, a False Discovery Rate (FDR; Benjamini and Hochberg 1995) correction for multiple comparisons was applied. We used an alpha level of 0.05 for all statistical tests.

### Pupil analyses

Raw pupil data were firstly filtered with a high-pass non causal Finite Impulse Response (FIR) filter of 1/12 Hz^1^ in order to remove slow oscillations in the pupil response not related to the task. We then extracted two types of segments: (i) from −100 to +1200 ms time-locked to stimulus onset and (ii) from −1400 to +400 ms time-locked to perceptual response onset. Individual segments were corrected with a 100 ms baseline. Specifically, we subtracted the average pupil size observed from −100 ms to 0 ms prior to stimulus onset for each trial from each time point of the corresponding trials. Blinks were linearly interpolated. The correct interpolation of blinks was verified through visual inspection of each segment. Visually inspecting the segment allowed us also to remove trials containing artifacts (e.g. trials containing blinks that were not correctly labeled and interpolated, epochs containing artifactual activity such as sudden spikes of pupil changes, epochs exhibiting pupil changes that exceeded a threshold of 700 units^2^ from the baseline). This led to the removal of an average (across participants) of two trials in the stimulus-locked segments (i.e. 0.35% of the trials) and to 8.64 trials in the response-locked epochs (i.e. 1.5% of all trials). Pupil segments were then down-sampled by averaging the pupil size of consecutive 100 ms time windows. From the clean segments, we computed pupil dilation velocity (*v* = Δ*s*/Δ*t*; where *s* is pupil size and *t* is time) since studies suggested that temporal derivatives of pupil size are more sensitive and reflect more closely the dynamics of arousal fluctuation (Reimer et al. 2014, 2016, McGinley et al. 2015, Okun et al. 2019, Crombie et al. 2021, Wang et al. 2022). Note that raw pupil size was also analyzed. Raw pupil data showed no significant correlation with confidence, neither with accuracy, nor with task difficulty. A graph of raw pupil size for the stimulus and response segment is reported in the supplementary material.

Pupil data were analyzed in two steps. Firstly, given that we had no prior assumptions regarding the timing exhibiting a possible relation between confidence and pupil, we performed a cluster-based permutation test comparing high confidence and low confidence trials. The aim of this first step was to isolate a temporal window where the pupil could dissociate low and high confidence trials. After isolating these temporal clusters, pupil data points within the time window were averaged and analyzed with linear mixed-effect models to evaluate their relationship to predictors of interest.

### Classification analyses

In a series of analyses, we used Linear Discriminant Analysis (LDA; cf. Carlson et al. 2003) to evaluate to what extent pupil velocity measured during stimulus presentation (hence prior to participants’ perceptual responses) could predict the individuals’ judgment of confidence. We then compared the prediction accuracy of the pupil classifier to the accuracy observed with a classifier predicting confidence judgments from individuals’ response times (i.e. confidence strongly correlates with perceptual decision latencies, notably the slower a perceptual decision the lower the confidence, cf. Mamassian 2016). This comparison would help us evaluate the quality of pupil-based classifier predictions with respect to a well-established proxy of confidence.

LDA classifiers were trained and tested for each time-point of the pupil velocity data to dissociate high and low confidence trials for each participant. Given that the stimulus-locked segment was not contaminated by blinks, we used this time window for the classification. The classification procedure implemented a Monte Carlo cross-validation method (Dubitzky et al. 2007). Notably, each classifier was trained on 90% of the available dataset and tested on each of the remaining trials. This procedure was repeated 1000 times. Each time, a random 90% of trials was used as a training set and the rest as a test set. It is important to stress that the number of trials were matched between high and low confidence on which the classifier was trained and tested to avoid classification biases. Classification accuracy was estimated by calculating for each time-point the proportion of trials that the classifier correctly identified as high or low confidence trials. The mean classification performance of the 1000 shuffling within each participant and time-point was taken as the classifier’s accuracy for that specific participant and time-point. For statistical analyses, chance level (a probability of 0.5) was subtracted from classification accuracy. Statistical significance (α = 0.05) was then calculated using a cluster-based permutation test performed on the resulting values. This test was chosen because we had no prior assumptions regarding the timing within the pupil segment exhibiting a possible relation between confidence and pupil. The same classification approach was used to classify high and low confidence trials from reaction times, except that for this variable the statistical significance was assessed with a non-parametric one-sample two-tailed Wilcoxon signed rank test on the resulting values (no cluster-based permutation test was performed here since reaction times do not reflect temporal series).

## Results

### Confidence

**Figure 2.**
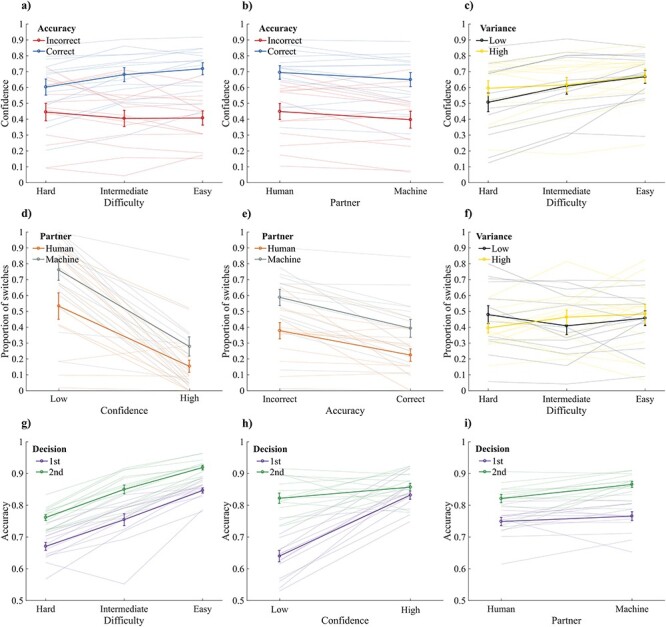
Plots showing dependent variables averaged across participants (thick lines), with error bars indicating the standard error of the means. Thin lines depict individual results. (a–c) Proportion of high confidence trials is shown as a function of accuracy and difficulty (a); as a function of accuracy and partner (b); and as a function of variability and difficulty (c). (d–f) Proportion of switches (in conflict trials) is shown as a function of partner and confidence (d); as a function of partner and accuracy (e); and as a function of stimulus variance and difficulty (f). (g–i) Proportion of correct responses is shown as a function of task difficulty and decision (g); as a function of confidence and decision (h); and as a function of decision and partner (i)

Logistic mixed effect models assessed the modulation of Difficulty, Variability, Partner, and Accuracy on confidence judgments. After addressing converging and singularity issues the final models for these analyses was the following: confidence ∼ difficulty * variability * partner * accuracy + (1 + difficulty + variability + partner + accuracy|| participants). A main effect of Difficulty was found [χ^2^(2) = 32.03, *P* < .001], with confidence increasing with the decrease of task difficulty (hard trials: M = 0.55, SD = 0.19; intermediate trials: M = 0.61, SD = 0.17; easy trials: M = 0.67, SD = 0.14). The significant interaction between Accuracy and Difficulty [χ^2^(2) = 32.14, *P* < .001; Fig. 2a] and simple main effect analyses showed that the main effect of difficulty was in particular driven by correct trials. In fact, confidence increased with the decrease of task difficulty only in correct response trials (*P* $ \le $ 0.010).

There was no main effect of Variability, but the interaction between Variability and Difficulty was significant [(χ^2^(2) = 12.03, *P* = .002; Fig. 2c]. Simple main effect analyses showed that confidence decreased in low Variability trials compared to high Variability trials only for the hard difficulty level (*P* = .003). Furthermore, confidence decreased in hard trials compared to both intermediate (*P* = .012) and easy trials (*P* = .011) only in the low Variability condition. Accordingly, the manipulation of stimulus variability had only partially the expected effect of modifying the confidence judgments.

As expected, a main effect of Accuracy on confidence was also found [χ^2^(1) = 111.06, *P* < .001], with lower confidence for incorrect (M = 0.42, SD = 0.18) compared to correct judgments (M = 0.67, SD = 0.16), showing that participants could evaluate the correctness of their responses. Interestingly, a main effect of Partner was also found [χ^2^(1) = 7.62, *P* = .006], with lower confidence in the blocks in which participants interacted with a machine (M = 0.59, SD = 0.17) compared to when they interacted with a human partner (M = 0.63, SD = 0.17), and irrespectively of whether the response was correct or incorrect (see Fig. 2b).

In sum, confidence strongly correlated with accuracy, showing that participants could clearly estimate the correctness of their perceptual decisions. Confidence was also affected by the identity of the interacting partner, while the overall accuracy (and reaction times; see supplementary materials) remained unaffected by the partner’s identity. Contrary to previous study (Desender et al. 2018), stimulus variability only mildly influenced confidence judgments, i.e. only when task difficulty was hard.

### Accuracy

Logistic mixed effect models assessed the impact of Partner, Variability, and Difficulty on the accuracy of the first perceptual decision. The final model after addressing convergence and singularity issues was the following: accuracy ∼ partner * variability * difficulty + (1 + partner + variability|| participants). The result showed only a main effect of Difficulty [χ^2^(2) = 222.75, *P* < .001], with accuracy increasing with the decrease in task difficulty (Fig. 2g). All other factors did not reach significance (*P* > 0.059). Accordingly, neither stimulus variability [χ^2^(1) = 3.56, *P* = .059] nor partner’s identity [χ^2^(1) = 1.88, *P* = .171] induced a change in the accuracy of the first perceptual decision.

After the first perceptual decision, participants reported their confidence and viewed the response of their partner. Hence, in a subsequent analysis, we investigated the modulation of these new variables on the accuracy of the second decision. The model included Decision (first, second), Partner (human, machine), and Confidence (low, high) and their interactions as fixed factors. The final model after addressing convergence and singularity issues was as follows: accuracy ∼ decision * partner * confidence + (1 + decision + partner + confidence + decision:confidence + partner:confidence|| participants). The analyses showed a main effect of Decision [χ^2^(1) = 52.87, *P* < .001], with higher accuracy in the second decisions, i.e. after viewing the partner’s report (accuracy first decision: M = 0.76, SD = 0.04; accuracy second decision: M = 0.84, SD = 0.03; Fig. 2g).

The main effect of Partner was also significant, notably accuracy increased when participants interacted with a machine compared to a human partner [χ^2^(1) = 8.27, *P* = .004]. A significant interaction between Decision and Partner [χ^2^(1) = 6.49, *P* = .011] and subsequent post-hoc tests showed that this main effect was driven by the fact that accuracy increased when participants interacted with a machine (M = 0.87, SD = 0.035) compared to when they interacted with a human partner (M = 0.82, SD = 0.049) only in the second decision (*P* < .001; Fig. 2i). However, no effect of Partner was observed for the first perceptual decision (see also previous analyses performed on the first perceptual decision only). This may suggest that participants tended to switch more often for the partner’s report when they thought they interacted with a machine compared to when they believed to interact with a human (see switch responses below).

Furthermore, we observed a main effect of Confidence [χ^2^(1) = 57.36, *P* < .001], with higher accuracy for high confident trials compared to low confident trials, and a significant interaction between Decision and Confidence [χ^2^(1) = 40.42, *P* < .001]. This interaction and subsequent post-hoc comparisons showed that accuracy was higher for the second decision compared to the first only when participants were not confident about their perceptual decision (*P* < .001; Fig. 2h, left side of the plot vs right side of the plot). This suggests that participants tended to switch for the partners’ response in particular when they were not confident in their decision, which improved their performances.

In sum, decision accuracy increased in the second decision more strongly when participants interacted with a machine compared to a human partner and when they had low confidence in their first decision. Importantly, the confidence results observed when participants interact with a machine (i.e. decrease in confidence) and when stimulus variability is low (decrease confidence in hard trials) cannot be explained by changes in accuracy.

### Switch responses

Logistic mixed-effect models were used to assess the factors influencing change of mind behavior. Given the multitude of factors (Confidence, Accuracy, Difficulty, Variability, and Partner), the extensive number of potential interactions for modeling switch responses, and the relatively small number of switch trials, we adopted a model comparison approach to determine the most relevant subset of fixed predictors for inclusion in the final model. We started with a full model (Model5) containing all fixed factors (Confidence, Accuracy, Difficulty, Variability, and Partner) and interactions. Subsequently, we compared this full model to a simpler version (Model4) from which we removed the quintuple interaction term. Model4 was then compared to a simpler model (Model3) where all quadruple interactions were omitted. This process continued iteratively, with each model being compared to a simpler version until we arrived at the most parsimonious model. The comparison led us to select a final model, which included Confidence, Accuracy, Difficulty, Variability, Partner, and all double interactions as fixed factors [switch responses ∼ confidence + accuracy + partner + variability + difficulty + variability:difficulty + variability:partner + variability:accuracy + variability:confidence + difficulty:partner + difficulty:accuracy + difficulty:confidence + partner:accuracy + partner:confidence + accuracy:confidence + (1 | participants)].

Importantly, the analyses of switch responses were performed only on conflict trials, i.e. trials where the participant selected a different response than the one chosen by the partner (in a total of 8064 trials, only once a participant decided to change his/her response even though there was no conflict with the partner’s response). With the final model we observed three main effects: a main effect of Partner [χ^2^(1) = 100.69, *P* < .001], with a larger proportion of switches when participants interacted with a machine (M = 54%, SD = 19%; Fig. 2e) compared to when they interacted with a human partner (M = 35%, SD = 17%); a main effect of Accuracy [χ^2^(1) = 8.57, *P* = .003], showing that participants switched more often for the partner’s report when they were incorrect (Fig. 2e, left side vs right side of the plot); and a large main effect of Confidence [χ^2^(1) = 382.45, *P* < .001], where participants switched more often when they were little confident regarding their response (Fig. 2d).

Interestingly, a Wilcoxon signed rank showed that participants tended to change their initial decision more often based on their confidence than their accuracy. Specifically, participants changed their response on average 49.2% more often when they were low confident compared to high confidence trials, while they changed their initial response 16.4% more often when they were incorrect compared to correct trials (see Table 1). These changes in switch responses were similar when considering the trials in which participants interacted with a machine and the trials where they interacted with a human partner, separately. Finally, no effect of difficulty on the proportion of switches was observed.

**Table 1.** Average proportion of switch responses (and standard deviation) for each confidence level as a function of accuracy

	Accuracy	
Confidence	Correct responses	Incorrect responses
High	0.15 (± 0.19)	0.23 (± 0.15)
Low	0.61 (± 0.31)	0.65 (± 0.26)

The logistic mixed-effect model also showed an interaction between Variability and Difficulty [χ^2^(2) = 9.54, *P* = .008]. Post-hoc pairwise comparisons contrasting the difference between the high and low Variability within the difference between hard and intermediate trials showed an opposite pattern of switch responses when looking at low and high variability trials (*P* = .015). Specifically, the proportion of switch responses increased in low-variability hard trials compared to low-variability intermediate trials. Conversely, in high-variability trials, an opposite pattern seemed to emerge: switch responses tended to decrease in high-variability hard trials compared to high-variability intermediate trials (see Fig. 2f). This finding aligns with the interaction observed between Variability and Difficulty in confidence judgments, where an increase in confidence was observed in hard trials compared to intermediate trials specifically in the low-variability condition, while accuracy remained constant. In essence, this may imply that low-variability hard trials led to decreased confidence, consequently prompting participants to change their initial decision. Finally, an interaction between Accuracy and Confidence was observed (χ^2^(1) = 3.99, *P* = .046), suggesting that the increase in switch responses observed in incorrect compared to correct trials was larger when participants were low confident compared to when they were high confident.

Additional analyses reported in the supplementary material further investigated the impact of Variability on confidence, accuracy, and switch responses. de Gardelle and Mamassian (2014), using a very similar stimulus as ours, observed a large variability in the effect of stimulus variance on confidence across participants: some participants were more confident with high variance stimuli while others felt more confident for low variance stimuli. To examine this inter-subject variability, we performed additional analyses in which accuracy, confidence, and switch responses were predicted by the “preferred” variability (i.e. the stimulus variance that on average led the participant to have more confidence in her/his decisions). These analyses showed that “preferred” variance impacted switch responses in a similar way as confidence but did not affect accuracy (see supplementary material).

### Pupillary response

**Figure 3.**
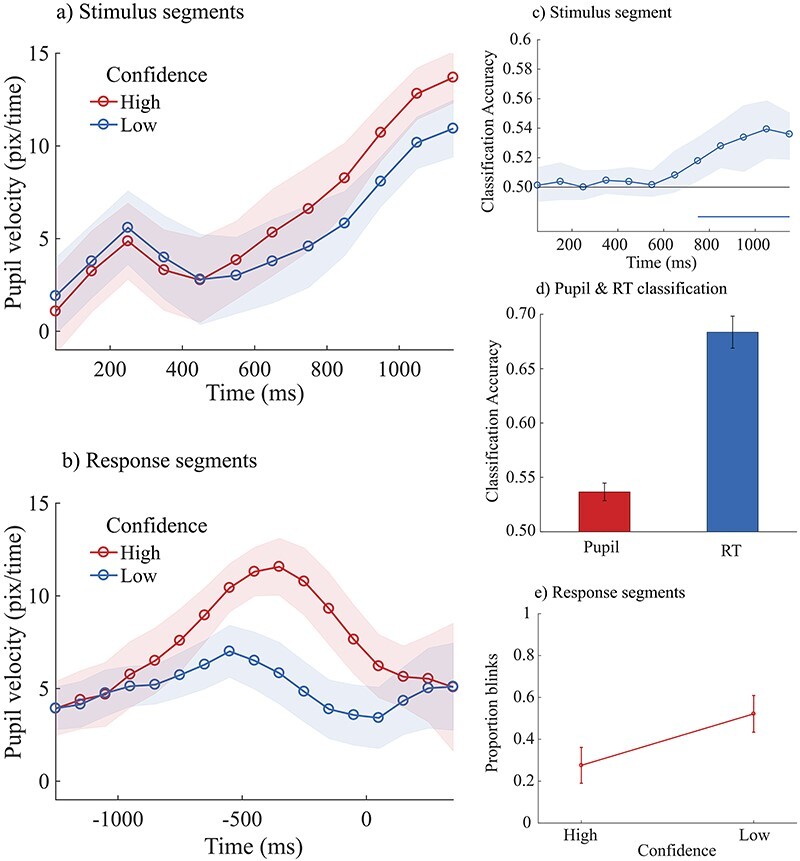
The plots 3a and 3b show the average pupil velocity for high and low confidence trials calculated across participants as a function of the time after stimulus onset (a), and the time around the perceptual decision (b). In plot 3a 0 ms is the onset of the RDKs and in plot 3b 0 ms is the onset of the perceptual decision. Unit in these time-series plots is pixels/time. With the EyeLink 1000 Plus, pupil area is calculated as the sum of the number of pixels inside the detected pupil contour. Hence, pupil dilation velocity was computed as v = Δs/Δt, where s is pupil size (in pixel) and *t* is time (in our case Δ*t* was 100 ms). Shaded areas represent ± 1 standard error of the mean. (c) Accuracy (proportion of correct classification) of a classifier dissociating high and low confidence trials from pupil velocity. The classifier was trained and tested on stimulus-locked segments (0 ms reflects the onset of the RDKs). The horizontal line below 0.5 accuracy indicates the time points with a classification accuracy that was significantly above chance level (50%). Shaded area represents bootstrapped 95% confidence intervals. (d) Best classification accuracy (across time) to discriminate high and low confidence from pupil velocity (in red) and from reaction times (in blue). Error bars represent standard errors. (e) Proportion of blinks that occurred in high and low confidence trials

A cluster-based permutation test showed that pupil velocity observed in high and low confidence trials differed within a time window going from 550 to 1150 ms (Fig. 3a). The pupil velocity observed within this time window was averaged and analyzed using linear mixed models. Given the large number of factors and interactions that could show a relationship with pupil dilation, a model comparison approach was used to remove from the model the factors that do not improve model prediction. We started with a complex model including Partner, Variability, Difficulty, Accuracy, Confidence, and their interactions as fixed effects. The model also included Switch response as predictor and the factor Participant as a random intercept [pupil velocity (stimulus segments) ∼ partner * variability * difficulty * accuracy * confidence + switch responses + (1 | participants)]. We then compared this model with a simpler model from which we removed all interaction terms. The comparison showed that including the interactions to the model did not significantly improve model fitting (χ^2^(41) = 52.39, *P* = .109). Consequently, we continued our analyses with the more parsimonious model described here as: pupil velocity (stimulus segments) ∼ partner + variability + difficulty + accuracy + confidence + switch responses + (1 | participants). Based on this mode, we then performed a backward elimination of the remaining fixed factors in order to find the subset of parameters leading to the best performing model. This led to a final model with only Accuracy and Confidence as fixed factors pupil velocity (stimulus segments) ∼ accuracy + confidence (1 | participants). The model showed that pupil velocity was higher for correct compared to incorrect responses [χ^2^(1) = 17.54, *P* < .001], and this effect was almost three times stronger when comparing high and low confidence [χ^2^(1) = 41.15, *P* < .001], with higher dilation velocity for high compared to low confidence judgments.

The same analyses on response-locked segments showed a difference between high and low confidence trials within a time window going from −550 to 50 ms (Fig. 3b). Pupil velocity observed within this time window was averaged and analyzed using the same approach described above. Including all the interaction terms in the model did not improve model fitting [χ^2^(41) = 41.75, *P* = .438]. Analyses were then continued with a parsimonious model [pupil velocity (response segments) ∼ partner + difficulty + confidence + accuracy + switch responses + (1 | participants)]. The backward elimination factors process on this model showed that all fixed factors contributed to pupil dynamics. Specifically, pupil dilation velocity was higher when participants interacted with a machine compared to when they interacted with a human partner [χ^2^(1) = 10.08, *P* = .002], when their response was correct compared to incorrect responses [χ^2^(1) = 18.94, *P* < .001], when they did not change their initial response [χ^2^(1) = 5.83, *P* = .016], when the task was easy compared to hard trials [χ^2^(2) = 6.41, *P* = .041], and finally more than the other factors, pupil dilation velocity was consistently linked to individuals’ confidence judgments [χ^2^(1) = 154.77, *P* < .001], with faster pupil dilation for high compared to low confidence judgments.

### Classification of pupil and RT

Similarly, to the analyses reported above, pupil-based classifiers could predict significantly above chance level individuals’ judgments of confidence within a time window going from 750 to 1150 ms after stimulus onset (Fig. 3c, see also supplementary material for additional classification analyses). The best performance (across time) of the pupil classifier was 54%. RT classifier could also predict significantly above chance level individual’s judgments of confidence with an average accuracy of 69% (Fig. 3d). A Wilcoxon Rank Sum Test showed that classification accuracy was higher with RT compared to the classification accuracy observed with pupil velocity [z(13) = 4.434, *P* < .001]. In additional analyses (not reported), we combined reaction times data and pupil data to assess whether classification accuracy would increase with both features. However, adding pupil data did not improve classification accuracy observed with reaction times alone.

### Blink data

Changes in pupil dilation may partly be caused by eye blinks (Lee et al. 2021). The response-locked segments contained eye blinks; hence, we decided to verify whether the proportion of blinks differed across conditions. The number of blinks observed in the response-locked epochs were analyzed with a mixed linear model including Partner, Difficulty, Confidence, Accuracy, and Switch response as fixed predictors and participants was included as a random intercept as follows: blinks ∼ partner + difficulty + confidence + accuracy + switch responses + (1 | participants) (i.e. the same final model used for pupil analyses in the same pupil segment). The number of blinks increased with incorrect compared to correct responses [χ^2^(1) = 7.45, *P* = .006]; it also increased when participants changed their initial response [χ^2^(1) = 14.80, *P* < .001] and when their confidence was low compared to high confidence trials [χ^2^(1) = 308.14, *P* < .001] (Fig. 3e).

## Discussion

This study aimed at contributing to: (i) the understanding of the impact of confidence in post-decisional behavior, (ii) exploring the link between pre-response pupil dilation and confidence, and (iii) confronting personal perceptual decisions with the ones of other humans and machines. Participants completed a two-alternative-forced-choice discrimination task where they had to identify the dot display containing a dot motion direction that was closer to the vertical axis. After their perceptual decision, they estimated how confident they were. They then viewed in different blocks the response of a human or a machine partner. They were told to evaluate the response of their partner and use it to improve their own accuracy on the perceptual task. Specifically, participants could either keep their initial perceptual decision or change it for the other option. Concomitantly, we recorded participants’ pupil dilation to investigate whether it was a good predictor of confidence and whether it was informative about individuals’ strategies to keep or change their initial perceptual response.

In agreement with past research on visual confidence, we observed that participants could evaluate the correctness of their visual decision and that confidence was sensitive to the quality of sensory information (cf. Mamassian 2016 for a review). Multilevel regression analyses showed that confidence better predicted changes of mind than perceptual accuracy or task difficulty. Specifically, participants changed their initial response approximately 49% of the time when they were not confident compared to when they were confident, while around 16% of the time when their response was incorrect compared to when it was correct. Importantly, this behavior was observed both when they interacted with a machine or a human partner. In addition, no modulation of task difficulty on the decision to keep or change initial responses was observed, reinforcing the idea that what matters for subsequent behavior is the subjective representation of decision uncertainty (i.e. confidence). This seems obvious considering the fact that the brain cannot have direct access to objective external information, and must also take into account other sources of information such as internal states and prior knowledge. Taken together, these findings suggest that subjective confidence estimates guide post-decisional behavior, more than perceptual accuracy or task difficulty.

In addition, we found that interacting with a machine or a human partner modulated confidence judgments without impacting decision accuracy. In particular, when participants interacted with a machine, they exhibited lower confidence judgments in their initial decision compared to when they interacted with a human partner. In line with recent models of metacognition (Fleming and Lau 2014, Mamassian 2016), this suggests that confidence judgments do not rely only on the quality of current sensory evidence, but also on additional pieces of information that can bias confidence evaluations (Shekhar and Rahnev 2020) such as prior beliefs or contextual information (Fleming et al. 2018, Marcke et al. 2024). Importantly, the decrease in confidence observed when interacting with a machine seemed to reflect a confidence bias rather than a change in metacognitive sensitivity. In fact, a similar decrease in confidence was observed for correct and incorrect responses. Hence, it is unlikely that participants’ change of mind was driven by a better use of machine advice on incorrect trials.

Furthermore, interacting with a machine not only decreased confidence but also increased participants’ tendency to change their initial perceptual response. This seems to suggest that confidence estimations as well as partner’s identity guided participants’ strategies to keep or change their initial perceptual judgments. A possibility is that, even though the performance accuracy of the two fictive partners was strictly the same across the task, participants may have had prior assumptions that the machine would be more reliable than a human on the task. Hence, to achieve better performances from their subjective perspective, participants may attribute less weight to the responses given by a human partner compared to those given by a machine partner while also being less confident in their initial responses when interacting with a machine partner, which may suggest an adaptive post-decisional strategy where the likelihood of a change of mind is mediated by both confidence (Mattingly et al. 2016) and the partner’s identity.

The influence of partner identity on change of mind appeared to be not mediated (at least not entirely) by confidence. This is suggested by the weaker effect of partner’s identity on confidence compared to its effect on change of mind (see Results and Fig. 2d & e). Furthermore, multilevel regression analysis revealed two independent main effects of partner identity and confidence on change of mind, suggesting that partner identity impacts behavior beyond the influence of confidence. Thus, these findings support the notion that the effect of partner identity on switch responses is not mediated (at least not entirely) by confidence and that both factors contribute independently to changes of mind. This interpretation is further supported by a mediation analysis reported in the supplementary material showing strong direct effects of confidence and the partner’s identity on change of mind a weaker indirect effect of the partner’s identity on change of mind via confidence.

It is important to underline that the direction of the effect of a partner’s identity on confidence and change of mind should be taken with caution, as it may originate from personal considerations and contextual factors. In line with this, recent studies showed that machines inspire overconfidence (Booth et al. 2017, Booth 2020) or mistrust (Nicodeme 2020, Seth and Kishore 2020, Lee and Rich 2021) depending on the situation. Furthermore, it has been shown that prior beliefs about a task could induce under- and overconfidence (Marcke et al. 2024), and that confidence plays a role in shaping certain aspects of decision-making behavior such as the confirmation bias (Rollwage et al. 2020) as well as driving post-decisional behaviors such as changes of mind (Rollwage et al. 2020, Pescetelli et al. 2021) or decision switch (Mattingly et al. 2016).

An alternative hypothesis is that the belief that the machine would perform better would lead participants to pay less attention to the perceptual task and strongly rely on the machine’s answers. However, this seems unlikely since no difference in accuracy in their initial perceptual decision was observed when participants interacted with a machine compared to when they interacted with a human partner, while stimuli were strictly identical in the two conditions (stimulus calibration using the staircase procedure was completed before the actual experimental task, the staircase trials did not include the partner’s identity manipulation, i.e. they consisted solely of the stimulus presentation followed by a perceptual response).

In summary, our study provides evidence that confidence predicts change of mind, and that partner’s identity influenced both confidence and changes of mind in an independent manner. Confidence seems to play a role in decisional behaviors, corroborating recent studies suggesting that confidence guides information seeking and learning (Meyniel et al. 2015, Guggenmos et al. 2016, Desender et al. 2018) as well as change of mind (Fleming et al. 2018, Rollwage et al. 2020, Pescetelli et al. 2021) and decision switch (Mattingly et al. 2016). Furthermore, it may be one of the mechanisms involved in human–human (Bang et al. 2017, De Martino et al. 2017) and human–machine interactions (Wright et al. 2020, Zhang et al. 2020). Joint decisions may sometimes require changes in personal opinion, so by weighting internal decisions and guiding decision-making behavior, perceptual confidence could play a key role in collective decision-making (Bahrami et al. 2010, Bang et al. 2014).

Contrary to previous studies, confidence was weakly modulated by the variability of the stimulus (Desender et al. 2018). Notably, we observed a change in confidence induced by stimulus variability only in hard trials, while accuracy remained unaffected by variance in those trials. However, similarly, switch responses appeared to be modulated by stimulus variance in hard trials, reinforcing the finding observed in multilevel regressions, suggesting that confidence, rather than first-order representations, better predicts changes of mind.

Regarding why confidence was modulated by stimulus variance only in hard trials, we posit that confidence judgments result from the weighted integration of various sources of information, including sensory evidence and contextual factors (e.g. prior beliefs and stimulus variance, Boldt et al. 2017, Schustek et al. 2019; Van Marcke et al. 2024). It is plausible that, in the current experiment, stimulus variance influenced confidence only when evidence-related signals were poor (i.e. in hard trials). In other words, stimulus variance carried low weight in a process integrating different cues for confidence when task difficulty was low, while when sensory evidence was very poor, participants relied on stimulus variance to judge confidence.

Regarding why participants were on average more confident in high variance trials compared to low variance trials, this could be explained in terms of inter-subject variability. In fact, this finding is not entirely new; de Gardelle et al. (2015), using a very similar stimulus as ours, showed that some participants were more confident with high variance stimuli while others were more confident with low variance stimuli. In other words, participants exhibited stable preferences regarding the stimulus variance (cf. de Gardelle et al. 2015). We investigated this notion further (see supplementary material). Specifically, we labelled trials based on the stimulus variance that participants preferred. If a participant on average rated his/her confidence higher in high variance trials than low variance trials, then we relabeled his/her high variance trials as “preferred” trials and the low variance as “non-preferred” trials. Conversely, if a participant on average rated his/her confidence higher in low variance trials than high variance trials, then we relabeled his/her low variance trials as “preferred” trials and the high variance as “non-preferred” trials. The analyses of the impact of preferred variance on confidence, accuracy, and switch responses showed that not surprisingly confidence increased with preferred compared to non-preferred variance. More interestingly, accuracy was not modulated by individuals’ preference, and switch responses followed a pattern similar to confidence; notably, switch responses increased in non-preferred trials compared to preferred trials. Taken together, these results suggest that while not affecting accuracy, the preferred stimulus variance affected confidence which in turn potentially modulated switch responses.

Decision-making is accompanied by broad neurophysiological changes of the body (O’Connell and Kelly 2021) including changes in eye pupil activity (Urai et al. 2017). In the present study we also investigated the relation between eye pupil changes and confidence. In particular, we analyzed pupil velocity since recent studies suggest that temporal derivative of pupil dilation reflects closely arousal fluctuations (Reimer et al. 2014, 2016, Okun et al. 2019, Crombie et al. 2021). The interest in using pupil dilation as a proxy of confidence relies on the fact that it could allow for the monitoring of individuals’ confidence and uncertainty online and through time without the need of collecting confidence judgments or measuring response times. This application can be important in different domains, including the field of human–human and human–machine interactions. There is indeed growing interest in neuroergonomics to monitor through time different cognitive states of operators while they interact with technology and their teammates (Dehais et al. 2017, 2020, Gramann et al. 2017), with the objective among others to conceive and evaluate new technology. In this context, confidence appears to be a critical phenomenon for optimal human–machine interactions (Lee and Moray 1992, Parasuraman et al. 1993), and to that extent, online access to operators’ confidence states could be particularly useful.

Interestingly, in agreement with previous studies (e.g. Lempert et al. 2015), confidence correlated with changes in pupil dilation dynamics. However, in addition to past research, our study showed that pre-response pupil dynamics predict confidence judgments. Specifically, we observed that during the presentation of the visual stimulus (when neither blinks nor responses could occur) pupil velocity was higher when the following response was correct or confident. Interestingly, pupil velocity did not correlate with the difficulty of the task. This dissociation together with the strong relation between pupil velocity and confidence (three times stronger than the relation between accuracy and pupil velocity) suggests that pupil velocity may be associated with the subjective evaluation of uncertainty rather than objective uncertainty, through short-term arousal fluctuations. A possible explanation for this finding would be that in confident trials participants allocated more strongly attentional resources to the stimulus compared to low confidence trials. However, if that was the case, we believe that pupil dynamics would then correlate more with correct responses than confidence. Our finding showed exactly the opposite: larger variations of pupil dynamics were observed when confidence varied rather than when accuracy varied. Secondly, pupil dilation dynamics differed between high- and low-confident trials when focusing only on correct trials (see supplementary materials). In agreement with confidence-based learning models, another possible explanation is that confidence has a mechanistic role in reward and value-based learning (Guggenmos et al. 2016, Ptasczynski et al. 2022). Interestingly, pupil dilatation, and more generally arousal, is associated with reward anticipation (Koelewijn et al. 2018, Schneider et al. 2018). We speculate that feeling confident in one’s own performance during a task may work as a reinforcing and rewarding signal influencing cognitive states, which would in turn modulate arousal-based pupil fluctuations.

Past research reported relations between pupil dilation and uncertainty post-response (Lempert et al. 2015, Urai et al. 2017, Balsdon et al. 2020). Our study provides new evidence on the link between pre-response pupil changes and confidence. In further analyses, we found that pupil dilations predict confidence only weakly (54% correct classification) compared to response latencies related to perceptual decisions (69% correct classification). Hence, reaction time remains a rather reliable proxy for confidence estimations, in line with previous literature (Kiani et al. 2014). In additional analyses (not reported), we combined reaction times data and pupil data to assess whether classification accuracy would increase with both features. However, adding pupil data did not improve the classification accuracy observed with reaction times alone. This may suggest that pupil data were redundant with respect to reaction times and that variation of reaction times and pupil dilation may be based on partially common mechanisms influencing confidence. In spite of the small contribution of pupil dilation for confidence classification, we believe that there is an intrinsic interest in classifying confidence based on pupil data only, since this measure does not require any overt behavior in order to potentially access the degree of confidence of a participant. However, classification accuracy remains very low for Brain Computer Interface. Further investigation should be encouraged to better understand the relationship between pupil and confidence and to improve classification accuracy of pupil-based confidence signals.

The analyses of pupil changes around the time of the perceptual response provided similar findings showing that, starting from −550 ms to +50 ms around the response, pupil velocity correlated strongly with confidence, accuracy, task difficulty, and participants’ tendency to change their initial response. However, this time window was contaminated by eye blinks which may have driven unwanted changes in pupil dilation velocity, thus making any conclusion regarding the changes in pupil velocity observed during this time period difficult to draw. In fact, we observed a larger proportion of blinks in hard, incorrect, and in low confidence trials. It is well known that stress, boredom, and fatigue can induce an increased blink rate (Tanaka and Yamaoka 1993, Barbato et al. 2000, Danckert et al. 2018). We speculate that the increase in eye blinks observed either in low confidence trials or in incorrect and hard trials is associated with momentary stress changes associated with low performance and task difficulty. Further studies should corroborate the relationship between confidence and eye blinks.

## Conclusions

In summary, our findings bring new evidence supporting that confidence as well as partner’s identity contribute to post-decisional behaviors and in particular change of mind during the interaction with another partner. Confidence would be a subjective evaluation of decision uncertainty, which integrates internal representations including current internal states and context-dependent beliefs about ourselves and others (Lebreton et al. 2019, Carlebach and Yeung 2023) in situations with incomplete knowledge (Khalvati et al. 2021). The role of confidence may be to weigh our decisions and perceptions and to modulate the likelihood of change of mind, allowing adaptation and learning, therefore contributing to decisional behaviors. Furthermore, the identity of the partner during collective decision-making is also a factor influencing confidence and also driving change of mind. Our results also provide new insight on the relation between eye pupil dilation and confidence. Crucially, eye pupil dynamics seem to provide online information about ongoing metacognitive processes and could also directly participate in decision-making and metacognition, in line with recent advances in the neurophysiology of perceptual decision-making (O’Connell and Kelly 2021).

## Supplementary Material

niae018_Supp

## Footnotes

1 The filter amplitude was determined as 1 divided by twice the average trial duration. On average a trial lasted 6 s (including the 0.5 s of ITI). This was done to remove slow oscillations that were not linked to our manipulations.

2 With the EyeLink 1000 Plus, pupil area is calculated as the sum of the number of pixels inside the detected pupil contour.
