# Supplementary material for: What the eyes, confidence, and partner’s identity can tell about change of mind
Source: Neurosci Conscious. 2024 May 7;2024(1):niae018. doi: 10.1093/nc/niae018 (PMC11077902; doi:10.1093/nc/niae018)
Supplement: niae018_Supp [file niae018_supp.zip › suppl_data/intco_Supplementary_Material.docx]

**Supplementary Material**

**What the eyes, confidence and partner’s identity can tell about change of mind**

Rémi Sanchez ^1, 2^, Anne-Catherine Tomei ^1, 2^, Pascal Mamassian ^4^,

Manuel Vidal ^2,^ * & Andrea Desantis ^1, 2, 3,^ *

*co-senior authors

^1^ Département Traitement de l’Information et Systèmes, ONERA, Salon-de-Provence, France

^2^ Institut de Neurosciences de la Timone (UMR 7289), CNRS and Aix-Marseille Université, Marseille, France

^3^ Integrative Neuroscience and Cognition Center (UMR 8002), CNRS and Université Paris Cité, Paris, France

^4^ Laboratoire des systèmes perceptifs, Département d’études cognitives, École normale supérieure, PSL University, CNRS, Paris, France.

**Results**

*Reaction Times.* A linear mixed effect model investigated the impact of Partner, Variance and Difficulty on reaction times observed for the first perceptual decision. The analyses showed a main effect of Difficulty (χ2(2) = 40.81, p < 0.001), with gradually faster perceptual responses with the decrease of task difficulty (hard trials: M = 1.896, SD = 0.189; intermediate trials: M = 1.850, SD = 0.187; easy trials: M = 1.790, SD = 0.172). In addition, we observed a significant interaction between Partner and Difficulty (χ2(2) = 6.93, p = 0.031) and between Variance and Difficulty (χ2(2) = 9.99, p < 0.007). None of the other comparisons were significant. Simple main effects analyses investigated the two significant interactions. In particular, we compared human and machine partner trials for each difficulty level. The analyses showed that RTs were faster only in hard trials (p = 0.025) when participants interacted with a human partner (M = 1.847, SD = 0.153) compared to when they interacted with a machine (M = 1.945, SD = 0.264). In other words, decisions in hard trials may have been taken faster when people interacted with a human compared to a machine partner. Finally, we compared low and high variance trials for each difficulty level. RTs were faster in hard trials (p = 0.003) with high variance (M = 1.852, SD = 0.166) compared hard trials with low variance (M = 1.940 SD = 0.220). Accordingly, decisions may have been taken faster when participants performed hard trials with high variance than with low variance.

**The impact of stimulus variance on confidence**

Regarding the question of why stimulus variance only mildly influenced confidence, we believe that different elements could explain this observation. Firstly, we believe that confidence judgments result from the weighted integration of different sources of information, including sensory evidence and contextual factors (e.g., prior beliefs and stimulus variance). It is plausible that in the current experiment, stimulus variance informed confidence only when evidence-related signals were poor (i.e., in hard trials). Regarding why we observed the higher confidence in high variable stimuli compared to low variable stimuli, this might be due to individuals’ preference. In fact, de Gardelle & Mamassian (2014) using a very similar stimulus as ours, observed a large variability across participants: some participants were more confident with high variance stimuli while others felt more confident for low variance stimuli.

To examine this inter-subject variability, we performed additional analyses. We labelled trials based on the stimulus variance participants preferred. Specifically, if a participant on average rated his/her confidence higher in high variance trials than low variance trials, then we relabeled his/her high variance trials as “preferred” trials and the low variance as “non-preferred” trials. Conversely, if a participant on average rated his/her confidence higher in low variance trials than high variance trials, then we relabeled his/her low variance trials as “preferred” trials and the high variance as “non-preferred” trials. Subsequently, we analyzed the accuracy, confidence and switch responses based on these new labels. A binomial (logistic) mixed model investigated the impact of Partner, Difficulty and Preference on accuracy. The model used after adjusting for convergence and singularity issues was: *accuracy ~ partner*preference*difficulty + (1 + partner + preference || subject)*. The analyses showed only a main effect of Difficulty on accuracy (χ2(2) = 222.04, p < 0.001), with accuracy increasing with the decrease of task difficulty. None of the other factors and interactions were significant (e.g., Partner: χ2(1) = 1.89, p = 0.169; and Preference: χ2(1) = 3.73, p = 0.054).

The same analyses on confidence judgments (*confidence ~ partner*preference*difficulty + (1 + partner + preference || subject)*) showed: a main effect of Partner (χ2(1) = 7.68, p = 0.006), with confidence decreasing when participants interacted with a Machine; a main effect of Difficulty (χ2(2) = 91.61, p < 0.001), with confidence decreasing with the increase of task difficulty; and not surprisingly a main effect of Preference (χ2(1) = 13.10, p < 0.001).

Finally, the same model applied to switch responses (*switch responses ~ partner*preference*difficulty + (1 + partner + preference || subject)*) showed a main effect of Partner (χ2(1) = 16.28, p < 0.001) and a main effect of preference (χ2(1) = 4.32, p < 0.038), with the proportion of switch responses increasing when participants interacted with a machine and when they were presented with a non-preferred variance.

Taken together these results suggest that while not affecting accuracy, the preferred stimulus variance affected confidence and in turn switch responses, by increasing confidence and decreasing switch responses compared to the non-preferred stimulus variance. However, note that when adding Confidence and Accuracy as predictors in the model, the main effect of Preference disappeared. Specifically, after addressing converging and singularity issues, and removing the interactions that did not contribute to the fitting of switch responses, the final model (*switch responses ~ preference + difficulty + partner + accuracy + confidence + (1 | subject)*) exhibited a main effect of Partner (χ2(1) = 98.56, p < 0.001), Accuracy (χ2(1) = 8.19, p < 0.004), and a very strong effect of Confidence (χ2(1) = 383.97, p < 0.001). The disappearance of the effect of Preference might be due to a redundancy with the factor Confidence.

**Raw pupil data**

The two graphs below depict raw pupil data as a function of time for high confident (red) and low confident (blue) trials. The left graph depicts changes in pupil size observed during stimulus presentation (0 ms reflect the onset of the RDKs) and the right graph shows pupil diameter around the perceptual decision (0 ms reflect the onset of the response), averaged across participants. No difference was observed in raw pupil data when comparing high and low confidence.


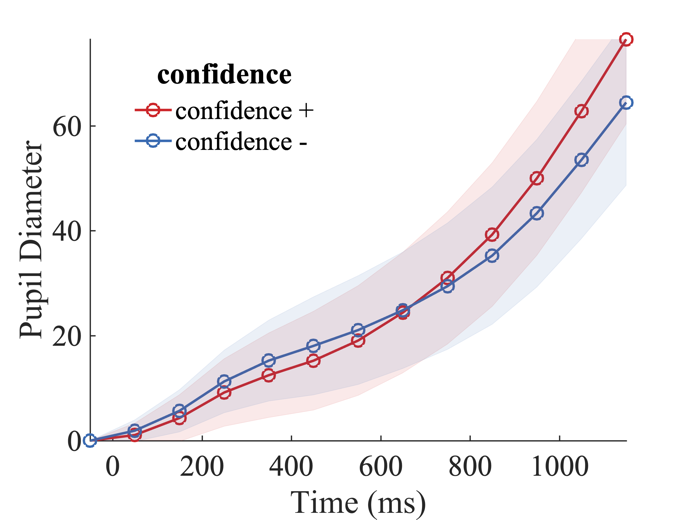

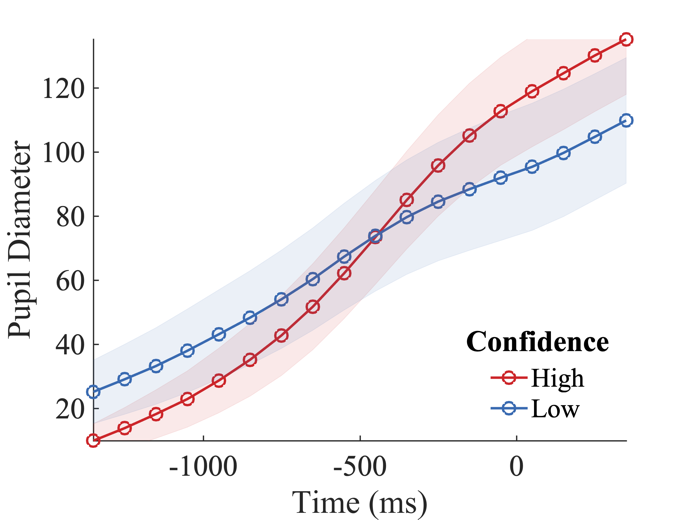


**Classification results**

The blue curve in Figure 2 depicts the accuracy (proportion of correct classification) of a classifier trained to differentiate, from pupil dilation, high and low confidence judgments in correct responses only. The classifier was trained and tested on stimulus-locked segments. The horizontal blue line depicts the time cluster with classification accuracy significantly above chance level (50%). Shaded area represents bootstrapped 95% confidence intervals. The red curve depicts the accuracy of a classifier trained to dissociate correct and incorrect responses in high confident trials only from pupil dilation. No significant time cluster was observed for the latter classifier.


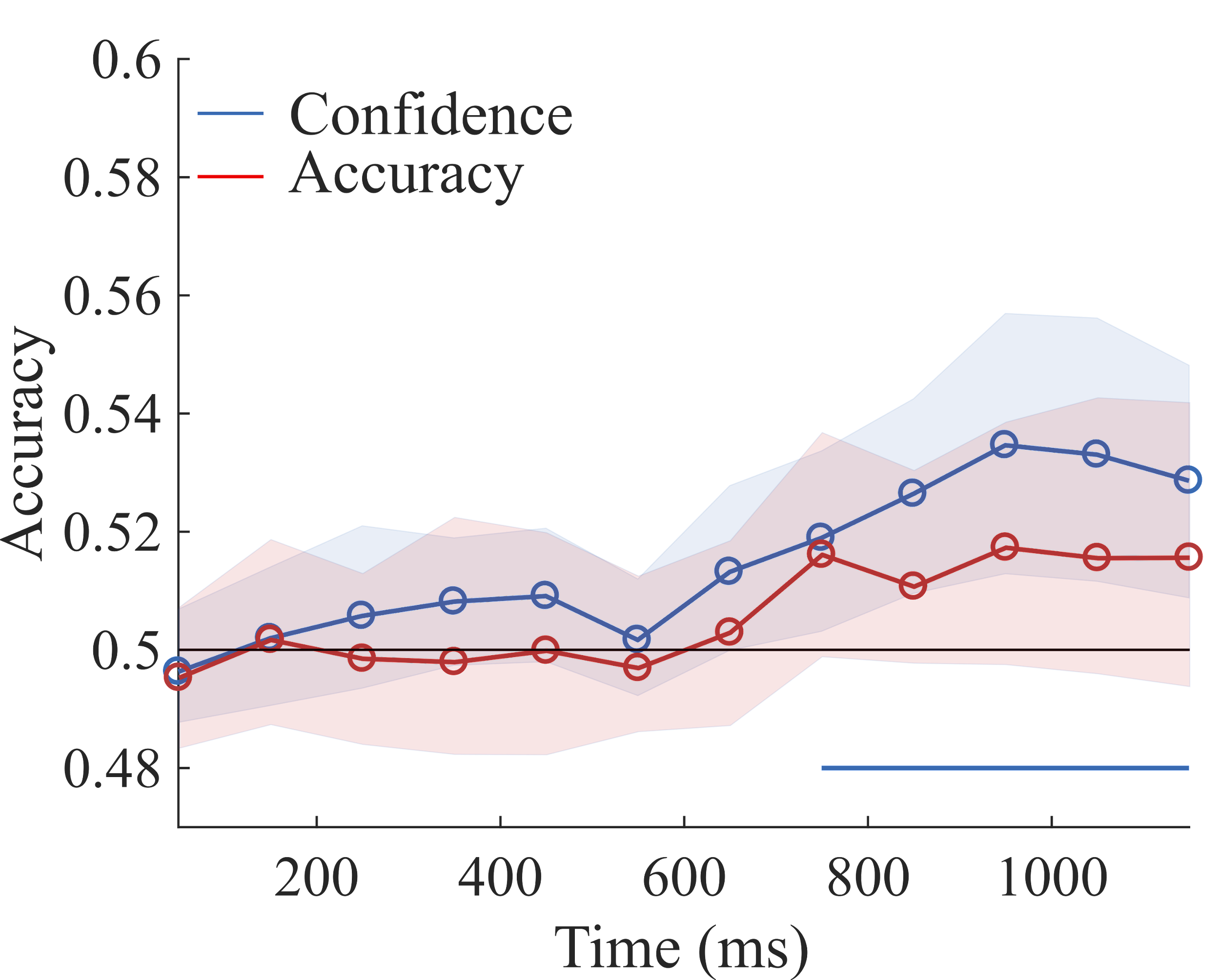


Figure 2

**Questionnaire**

At the end of the experiment, each participant was asked to answer a series of questions (Table 1) addressing his/her interaction with the computer and human partner. The main objective of this questionnaire was to evaluate whether participants questioned the fact that they were really viewing the responses or another participant or a machine-learning program. From the questionnaire and a short debriefing, participants trusted the scenario we created for these experiments. About 56% of participants felt that the computer partner performed better than the human partner and they felt they used the former's responses more often. About 31% of participants said they could not see or be able to differentiate the performance of the two types of partners.

**Mediation analysis**

We used the package Lavaan to perform mediation analyses and to investigate whether partner identity impact switch responses through confidence judgments. We defined the mediation model as follows:

mediation_model <- '

*# Direct effects*

confidence ~ a * partner

switch_response ~ c * partner + b * confidence

*# Indirect effect (a * b)*

indirect := a * b

*# Total effect (c + indirect)*

total := c + indirect

The analyses showed a significant direct effect of partner on confidence (z-value = 3.911, p < 0.001), a significant direct effect of partner on switch responses (z-value = 5.495, p < 0.001), and a larger significant direct effect of confidence on switch responses (z-value = 27.620, p < 0.001). The estimated indirect effect of partner on switch responses through confidence was also significant (z-value = 3.872, p < 0.001) but weaker compared to the other direct effects.

Table1. Questionnaire participants filled at the end of the experiment with its *translation in english*.

| **Questions** | **Oui *Yes*** | **Non**  ***No*** | **Je ne sais pas**  ***I don't know*** |
| --- | --- | --- | --- |
| Avez-vous utilisé les réponses du partenaire humain pour modifier vos choix?  *Did you use the human partner's responses to change your choices?* |  |  |  |
| Avez-vous utilisé les réponses du partenaire ordinateur pour modifier vos choix?  *Did you use the computer partner's responses to change your choices?* |  |  |  |
| Pensez-vous que l'un des partenaires était meilleur que l'autre?  *Do you think one partner was better than the other?* |  |  |  |
| Pensez-vous que le partenaire ordinateur était meilleur que le partenaire humain?  *Do you think that the computer partner was better than the human partner?* |  |  |  |
| Avez-vous utilisé davantage les réponses d'un partenaire par rapport à l'autre, pour modifier vos choix?  *Did you use more often the responses of one partner than the other to change your choices?* |  |  |  |
| Aviez-vous toujours en tête avec quel type de participant vous interagissiez, humain ou ordinateur?  *Did you always have in mind what type of participant you were interacting with, human or computer?* |  |  |  |
